# The NHS visitor and migrant cost recovery programme – a threat to health?

**DOI:** 10.1186/s12889-020-08524-9

**Published:** 2020-04-20

**Authors:** J. L. Potter, M. Burman, C. D. Tweed, D. Vaghela, H. Kunst, D. Swinglehurst, C. J. Griffiths

**Affiliations:** 1 Centre for Primary Care and Public Health, Blizard Institute, Queen Mary University of London, London, England; 2 MRC Clinical Trials Unit, University College London, London, England

**Keywords:** Policy, Healthcare access, tuberculosis, Migrant health

## Abstract

**Background:**

In April 2014 the UK government launched the ‘NHS Visitor and Migrant Cost Recovery Programme Implementation Plan’ which set out a series of policy changes to recoup costs from ‘chargeable’ (largely non-UK born) patients. In England, approximately 75% of tuberculosis (TB) cases occur in people born abroad. Delays in TB treatment increase risk of morbidity, mortality and transmission in the community. We investigated whether diagnostic delay has increased since the Cost Recovery Programme (CRP) was introduced.

**Methods:**

There were 3342 adult TB cases notified on the London TB Register across Barts Health NHS Trust between 1st January 2011 and 31st December 2016. Cases with missing relevant information were excluded. The median time between symptom onset and treatment initiation before and after the CRP was calculated according to birthplace and compared using the Mann Whitney test. Delayed diagnosis was considered greater or equal to median time to treatment for all patients (79 days). Univariable logistic regression was used to manually select exposure variables for inclusion in a multivariable model to test the association between diagnostic delay and the implementation of the CRP.

**Results:**

We included 2237 TB cases. Among non-UK born patients, median time-to-treatment increased from 69 days to 89 days following introduction of CRP (*p* < 0.001). Median time-to-treatment also increased for the UK-born population from 75.5 days to 89.5 days (*p* = 0.307). The multivariable logistic regression model showed non-UK born patients were more likely to have a delay in diagnosis after the CRP (adjOR 1.37, 95% CI 1.13–1.66, *p* value 0.001).

**Conclusion:**

Since the introduction of the CRP there has been a significant delay for TB treatment among non-UK born patients. Further research exploring the effect of policies restricting access to healthcare for migrants is urgently needed if we wish to eliminate TB nationally.

## Background

In 2017, 1.6 million people died from world’s deadliest, yet curable, infectious disease - tuberculosis (TB). TB is an infectious disease transmitted by coughing. Whilst some people become unwell straight away, some carry the infection with no symptoms for many years, this is termed latent TB infection (LTBI). People living in countries with a high incidence of TB are at higher risk of contracting the infection that people living in places where TB is less common.

TB is unequally distributed; the world’s poorest people bear a disproportionate burden of disease [1]. In high-income, low-incidence settings such as the UK, the majority of active TB cases occur among migrants coming from countries where TB is more common [2]. Countries of origin amongst foreign-born patients reflect both migrant flows and the global distribution of TB, with the highest numbers of cases amongst people from high TB-incidence regions such as South Asia and Sub-Saharan Africa. In the UK, evidence suggests that TB in the migrant population is largely due to reactivation of latent TB infection, originally acquired abroad [3, 4].

There is no internationally recognised definition of the term migrant and in other contexts it can be used to refer to an individual who has moved away from their usual place of residence to another region within a country or across international borders to another country. In this paper the term migrant is used to refer to an international migrant. With respect to this study this includes any person living within the UK who was not originally born there. This definition incorporates many different categories of migrant including but not limited to so-called ‘economic migrants’, refugees, asylum-seekers, failed asylum-seekers and undocumented migrants.

Strategies to control TB centre around three key principles: early diagnosis; effective treatment; and preventative interventions for those at high risk (including both treatment of latent TB infection (LTBI) and BCG vaccination) [5]. The insidious, non-specific nature of symptoms associated with TB, such as fevers and weight loss, often translate into circuitous journeys to diagnosis, involving many different healthcare providers [6, 7]. The window of time that elapses between symptom onset and treatment is critical. If the time-to-diagnosis increases, the individual concerned is exposed to higher risk of morbidity and mortality, and, for infectious cases, the wider public is exposed to the risk of disease transmission [8–10].

Recent theoretical frameworks to describe healthcare access account for a complex and dynamic process, shaped by social interactions within particular contexts. ‘Candidacy’, for example, has frequently been employed to understand individuals’ experiences of accessing healthcare and describes “the ways in which people’s eligibility for medical attention and intervention is jointly negotiated between individuals and health services” [11, 12]. Included in this concept is the influence of “policy imperatives”.

The UK National Health Service (NHS) is a tax-funded system designed to provide care, free at the point of delivery. Eligibility for free NHS healthcare is determined by the test of ‘ordinary residence’; tourists and some migrants are required to pay for care [13]. Over recent years one policy imperative has been to ensure that individuals not ordinarily resident in the UK make a ‘fair contribution’ to the NHS. Changes in the Immigration Acts 2014 and 2016 have provided the legislative framework which has resulted in tightening of the definition of ordinary residence – the test of eligibility for free NHS care - and strengthening of mechanisms for cost recovery from chargeable individuals. Experiences of people from the Windrush generation published in mainstream media between 2017 and 2018 illuminated the broader impact of these policies: Individuals denied access to treatment such as chemotherapy because they could not demonstrate eligibility for free NHS care.

The health service in the UK is devolved; the policies discussed in this paper are specific to England. The NHS Visitor and Migrant Cost Recovery Programme (CRP), introduced in April 2014, was designed to ensure migrants in England who are otherwise ineligible for free NHS care make a ‘fair contribution’ [14] to the cost of running the NHS. Policies such as this may play an important role in shaping patients’ experiences of healthcare access [11, 12, 15, 16].

**Fig. 1:**
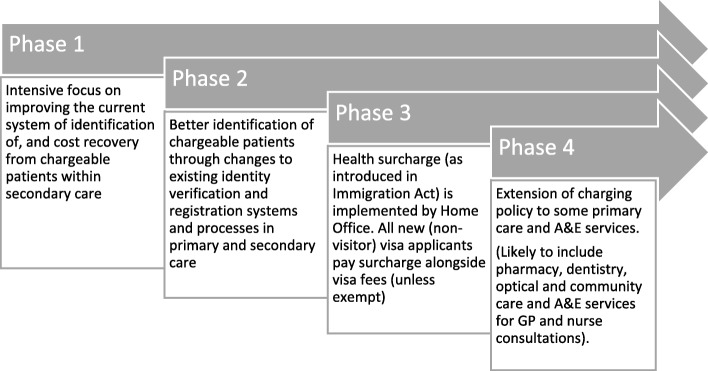
Phased approach reproduced from the Visitor & Migrant Cost Recovery Programme Implementation Plan 2014–2016

The CRP was supported by changes to legislation including the Immigration Act 2014 which increased the recoverable cost of treatment from 100% of tariff to 150% for ineligible residents from outside the European Economic Area. The ‘roll-out’ incorporated a range of new activities: strengthening of overseas visitor manager teams; more targeted strategies for the identification of ineligible service-users; introduction of punitive sanctions for NHS Trusts who failed to implement CRP measures robustly; and incentives enabling the recovery of increased tariffs from patients [17] (Fig. 1). House of Commons transcripts on this issue at the time [18], along with supporting policy documents [14] detail the need for a ‘change in culture’ such that determinations of eligibility and charging should become business-as-usual within the NHS.

Barts Health NHS Trust serves three East London boroughs and treats over 500 patients with a diagnosis of active TB each year. This represents approximately 10% of cases in England. National data is collected on all TB cases and includes: time between symptom onset and start of treatment; country of birth; and year of entry to the UK. Beyond these, migrant subgroups are not further classified and, in particular, immigration status and eligibility for free NHS care are not recorded. This study uses routinely collected surveillance data to examine time to treatment onset among UK-born and non-UK born populations in this region, before and after implementation of the CRP.

## Methods

The London TB Register (LTBR) is an online database for the statutory recording of notified cases of TB, as entered by clinical staff. The database contains details of patient ethnicity; gender; age; place of birth; length of time resident in the UK; date of symptom onset; date TB treatment started; occupation; and other medical history. We collected patient data from the LTBR for TB cases notified across Barts Health NHS Trust between 2011 and 2016 inclusive. We included all adult cases (≥18 years old) that did not have any missing relevant data.

We manually reviewed the dataset and categorised patients for the analysis as follows. Time in the UK was categorised as 0–1 years, 2–5 years, 6–10 years, or 11+ years. Patient occupation was classified using Office of National Statistics guidance as “managerial”, “intermediate”, “unskilled”, “unemployed”, “housewife/ house husband”, “student”, “unknown”, or “retired” [19]. We identified patients as having either “no social risk factors” or “at least one social risk factor” based on the additional information on the LTBR. Social risk factors are relevant to adherence rather than risk of infection, although there is clearly some overlap, and included: history of incarceration; drug or alcohol dependence; homelessness; and mental health concerns.

We identified patients as either “pre-CRP” or “post-CRP” according to the date they started their treatment for TB (before or after 1st April 2014, respectively). April 1st 2014 was the date provided in the policy. It signifies a conservative estimate of the beginning of the implementation of a complex series of policies and practices designed to restrict healthcare access for some groups of people not born in the UK. We also categorised patients as either “UK-born” or “not UK-born” based on place of birth data in the LTBR. In 59 cases, there was no data on treatment start date recorded in the LTBR database. For these cases we used the date the patient was first seen in TB clinic as a proxy measure.

### Statistical analysis

All data handling and statistical analyses were performed using Stata version 15 (StataCorp, Texas). Statistical significance was set at 5% for all tests. The median time to treatment was selected as the outcome of interest due to a combination of robustness against outliers, a lack of normal distribution in the time to treatment data, and simplicity of understanding. The Chi square test was used to compare the proportion of patients UK-born or not UK-born with each baseline characteristic when grouped as pre-CRP and post-CRP.

The time-to-treatment was calculated for all patients as the number of days between the date of symptom onset and the date of starting treatment. The Mann-Whitney test was used to compare the median time to treatment for all patients before and after the CRP. The difference in time to treatment before and after the CRP was then compared for patients according to their status as ‘UK-born’ or ‘born outside the UK’, again using the Mann-Whitney test.

We defined ‘delayed diagnosis’ as any patient with a time to treatment greater than the median value for all patients. We defined delay in this way because there is no established definition of what constitutes a delayed diagnosis of TB available in the literature [8]. In addition healthcare access is highly contextual and so applying alternative conceptions of delay from other regions or countries would not be appropriate. The proportion of all patients with a ‘delayed diagnosis’ before and after the CRP was investigated using the Chi square test, followed by patients according to their UK-born status. Assumptions relating to the Chi-square test, including minimum number for expected value per cell, were found to have been met.

Logistic regression was used to investigate the relationship between diagnostic delay (as a binary outcome) and whether date of diagnosis was before or after the CRP for patients born outside the UK. Variables were manually selected based on significance level in a univariable model (*p* value < 0.10) with the exposure (treatment before or after the CRP) and outcome variables (delay in diagnosis). These were included, along with sex and age, in a final multivariable logistic regression model testing the association between delay in diagnosis and introduction of the CRP to account for potential confounding. The regression model was tested using the Hosmer-Lemeshow test, and the minimum number of measurements was thought to have been reached in each of the two patient groups based on the number of variables being tested.

## Results

A total of 3342 cases were added to the LTBR between January 2011 and December 2016. There were 2237 cases were included in the final analysis (see Table 1). The median time to treatment for all included patients was 79 days. In the time period prior to the introduction of the CRP the median time to treatment was 70 days for all patients and this increased to 89 days after the CRP was introduced (*p* value< 0.001).

**Table 1:** Baseline characteristics for patients diagnosed before and after CRP based on UK-born status

	Pre-CRP		Post-CRP	
	*UK born*	*Non-UK born*	*UK born*	*Non-UK born*
Total cases	166	1037	170	864
Median age (IQR)	32.0 (23–46)	32.0 (26–45)	33.5 (25–47)	37.0 (29–49)
Sex				
Female (%)	43.5	36.9	32.5	37.5
Male (%)	56.5	63.1	77.5	62.5
Pulmonary TB Cases (% Smear Positive)	109 (45.87)	428 (36.5)	101 (31.7)	367 (33.9)
Extra-pulmonary TB cases (% of total cases)	61 (35.6)	609 (58.7)	65 (39.2)	497 (57.5)
% with ≥1 social risk factors	20.0	6.5	18.7	7.6

Of note, significantly more UK-born patients had more than one social risk factor compared with non-UK born patients (Chi-squared *p* < 0.001). There was also a significant increase in the number of years migrant patients had been living in the UK before their diagnosis with TB pre-CRP (10 years) and post-CRP (14.8 years, Chi-squared *p* value p < 0.001).

**Fig. 2:**
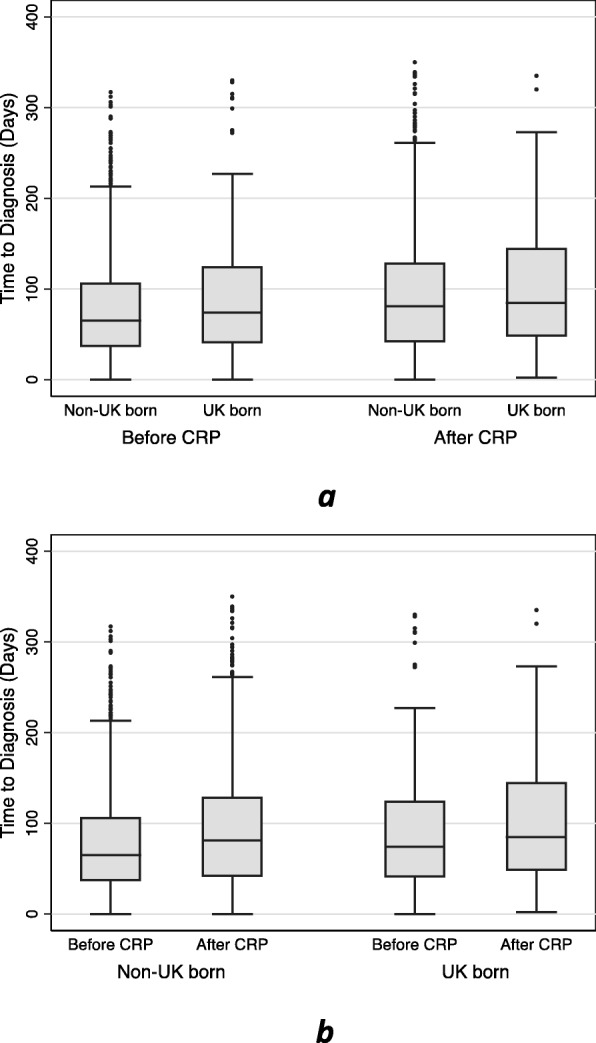
Boxplot showing time to diagnosis (in days) by a) whether patients were treated before or after the introduction of the Cost Recovery Program (CRP), sub-catergorised by place of birth and by b) place of birth, before and after the introduction of the CRP. Times to diagnosis above 350 days have not been displayed. Time to diagnosis: days from onset of symptoms to starting treatment

There was a significant increase in the time-to-treatment for non-UK born patients following the implementation of the CRP (see Table 2 and Fig. 2). Pre-CRP, the median time to treatment was 69 days in this group, increasing to 89 days post-CRP (*p* value < 0.0001). There was also an increase in the median time to treatment for UK-born patients post-CRP (75.5 days vs 89.5 days) but this increase was not significant (*p* = 0.307). The proportion of patients categorised as having a delayed diagnosis according to place of birth and CRP timing are shown in Table 3.

**Table 2:** Median time to diagnosis before/after the CRP for all patients, and then stratified by UK-born status

Patient group	Median time to diagnosis (days)	*p* value
All		
*Before-CRP*	70	0.001
*After-CRP*	89	
UK-born		
*Before-CRP*	75.5	0.307
*After-CRP*	89.5	
Non-UK Born		
*Before-CRP*	69	< 0.001
*After-CRP*	89	

*CRP*  Cost Recovery Program

**Table 3:** Proportions of UK-born and Non-UK born patients with a recorded delay in diagnosis before and after the introduction of the CRP. Delay in diagnosis is greater than or equal to median time (79 days) for all patients. Row percentages shown for proportions of patients with diagnostic delay

Place of birth	Delay in Diagnosis	Delay (Row %)	No Delay (Row %)	*P* value
*UK-Born*	Pre-CRP	82 (48.2)	88 (51.8)	0.227
	Post-CRP	91 (54.8)	75 (45.2)	
*Non-UK Born*	Pre-CRP	462 (44.6)	575 (55.5)	< 0.001
	Post-CRP	477 (55.2)	387 (44.8)	

After univariable analysis the following variables had associated *p* values < 0.10 and were included as exposure variables to adjust for confounding in the final multivariable logistic regression model due to a *p* value > 0.10: patient age; gender; time in the UK (as a categorical variable); and the presence of one or more social risk factors for TB. We excluded the following variables from the model: being born in an English-speaking country, site of TB disease, previous TB and being a healthcare worker because no association was found on univariate analysis.

Table 4 shows the results from the multivariable logistic regression analysis for patients born outside the UK. There was a significant association with treatment delay from the onset of symptoms in a multivariable logistic regression model (adjusted OR 1.37, 95% CI 1.13–1.66, *p* value < 0.01) among this group. Increasing age was also positively associated with treatment delay for patients born outside the UK. Conversely, the presence of one or more social risk factors was negatively associated with a treatment delay. The Hosmer-Lemeshow goodness-of-fit test was not statistically significant (*p* = 0.4596) suggesting the model is consistent and adequate to explain the observed outcome.

**Table 4:** Results of the multivariable logistic regression model with exposure variables shown as rows. Odds ratios relate to the binary outcome variable of diagnostic delay (greater than or equal to median time to diagnosis). Results shown are only for patients born outside the UK. Social risk factors for TB included history of alcohol dependence, recreational drug use, homelessness or imprisonment

	Adjusted Odds Ratio (95% confidence interval)	*p* value
Treatment after CRP	1.37 (1.13–1.66)	0.001
Age	1.01 (1.00–1.02)	0.010
Sex	1.13 (0.92–1.40)	0.245
Time in the UK		
0–1 year	REFERENCE	n/a
2–5 years	1.05 (0.77–1.43)	0.779
6–10 years	0.96 (0.68–1.36)	0.834
11+ years	1.14 (0.81–1.59)	0.452
Occupation		
managerial/professional	REFERENCE	n/a
Intermediate	0.86 (0.54–1.35)	0.512
Unskilled	0.82 (0.52–1.30)	0.405
Unemployed	0.73 (0.46–1.16)	0.182
Housewife/Househusband	0.74 (0.44–1.24)	0.253
Student	0.62 (0.38–1.02)	0.061
Unknown	0.59 (0.32–1.09)	0.089
Retired	0.43 (0.23–0.78)	0.006
1 or more social risk factor for TB	0.76 (0.61–0.96)	0.019

## Discussion

In 2016 the average time between symptom onset and starting treatment for pulmonary TB in England was 77 days [2]. A key aim of Public Health England and NHS England’s Collaborative Tuberculosis Strategy is to “improve access to services and ensure early diagnosis” [20]. This study has identified that, since the implementation of the CRP, migrants in East London are more likely to experience delay in treatment of their TB. This is an important finding since the longer the delay in diagnosing TB, the greater the morbidity [21] and mortality [22] for the individual, and the greater the risk of transmission in the community [23].

There are a number of potential mechanisms at play which may be relevant in the association between the experiences of migrants accessing healthcare and health policies that restrict access to care based on immigration status [11, 12]. There is already evidence to show that migrants in the UK are often not aware of their entitlements to care [24]. This is further complicated by recent legislative changes that have altered who is eligible for care and who is not, including the introduction of an immigration health surcharge that accompanies visa applications [25]. Previous research has shown patients’ concerns about being charged for care delay health-seeking, even before a diagnosis has been made [26–28]. This is important because diagnosis and treatment of TB, like many other infectious diseases, is exempt from charging in the UK, regardless of immigration status [29]. Crucially, however, patients present with undifferentiated symptoms - not a diagnosis - and many may be unaware of the details of the regulations [30].

Recent evidence suggests the CRP is just one policy among several which constitute a broader ‘hostile environment’ aimed at people living in the UK illegally [31]. For example a data-sharing agreement between NHS Digital and the Home Office [32, 33] requires that NHS staff report people with an outstanding bill exceeding £500 to the Home Office. Significant sharing of patients’ demographic information between the NHS and the Home Office has been reported [34].

The ‘hostile environment’ is an important socio-political context within which people make decisions about seeking help, including decisions regarding whether and how they identify themselves as a candidate for health care [35]. This effect may be independent of their legal eligibility for free care. Fear as a deterrent to healthcare access among migrants has been well documented in several countries including the UK [24, 36]. In 2012 the government made clear their explicit intention to create a “really hostile environment” for those living in the UK without the legal right to do so [37]. Aside from the CRP, other measures included: a rise in immigration raids; the prospect of unlimited detention; the threat to health and even life that immigration detention poses [38, 39]; school meals withheld because of parents’ immigration status [40], restrictions to the housing rental market, driving licenses and bank accounts in similar ways to the restrictions applied to the NHS [41]. The influence of these other ‘hostile environment’ policies have not been accounted for within this study but may have contributed to the overall findings.

There are other contemporaneous policy-related contexts which may be relevant to the increase in time to treatment reported in this study. Access to translation services have changed due to imposed cuts under conditions of austerity. Across the NHS, staffing levels decreased and waiting times for hospital care increased during the study period amidst concerns of an NHS ‘in crisis’ [42]. It is important to consider whether local changes in service provision may have impacted the results. The study data is collected from three London boroughs each served by a hospital with an A&E and local respiratory and TB services. Whilst there was an increased focus on TB among migrants during the study period, including education and awareness raising in primary care and local communities, no other major service changes occurred during this time. Finally, as with all observational analyses, causal associations cannot be inferred between the implementation of the CRP and the significant increase in the time to diagnosis.

Our study shows the UK-born population experienced a non-significant increase in the time to treatment of TB. A significantly higher proportion of UK-born patients had one or more social risk factors compared with those not born in the UK. This is reflective of national data [2]. Therefore the UK-born population is potentially more vulnerable to the effects of austerity; health suffers whilst individuals manage other competing priorities such as employment, housing, and limited income (earned or through welfare) restricting their means to access care [43]. This would result in an underestimation of effect. Conversely, clinicians’ sensitivity to TB as a differential diagnosis is likely to be heightened among patients with particular risk factors such as homelessness. However, not being born in the UK – particularly individuals from high TB incidence countries – is also likely to increase alertness amongst clinicians to the possibility of a TB diagnosis.

Nevertheless, these factors do not explain why only the non-UK born population experienced a significant delay in time to diagnosis and treatment following the introduction of the CRP whilst the UK-born population did not. Of note, migrants diagnosed with TB after 2014 had been in the UK significantly longer than those diagnosed before. There may be a number of reasons for this such as changes to immigration policies and the introduction of pre-entry TB screening [44]. Nonetheless, ‘newness’ of migrants has been associated with increased difficulty accessing care [35, 45] potentially resulting in an under estimation of the effect size.

Whilst the study area is geographically small, it accounts for approximately 10% of all cases of TB in the UK during the research period [2]. TB in the UK is largely focused in urban areas with large migrant communities. Thus it is possible our findings may be applicable to other areas within England which have similar migrant populations and have been subject to similar policies designed to restrict access to healthcare for some migrants and visitors. However, this study does not demonstrate causality and further research is required to examine the nature of the relationship between different categories of migrant, their eligibility for free NHS care and the complex and evolving arena of laws, policies and practices which shape access to TB treatment.

There are a number of limitations to the study. This paper does not claim causality but nevertheless demonstrates an important association that warrants further investigation. The ability to communicate in the language of the host country has been shown to affect healthcare access as well as the quality of care received by migrants [46], however English language ability is not routinely collected. Immigration status has also been shown to affect healthcare access [47] but is not routinely collected. It was therefore not possible to differentiate between migrants eligible for free NHS care and those who are not. Socio-economic status was determined through a proxy measure – occupational status – which has well-documented limitations [48]. A binary cut-off before and after the CRP does not reflect the reality of a policy which was rolled out over subsequent years. The unbalanced nature of the numbers in the UK-born and non-UK-born groups should not have introduced bias to the tests for significance used here but could have had an impact on the power of these tests to detect significant differences. One potential way to try to address this asymmetry in future work could be to employ matching, however careful thought would have to go into the selection of matching criteria. There are other techniques that could have been used to analyse the data that would account for the longitudinal nature the dataset, such as an interrupted time series analysis. However, this would introduce additional assumptions such as linearity of the data, predictable change in time-varying external factors, and autocorrelation. The findings presented here merely suggest an association with time as a binary variable using a simple test of proportion.

## Conclusion

This research demonstrates a significant association between diagnostic delay for patients with TB who were not born in the UK and policies which restrict healthcare access to visitors and migrants not entitled to free NHS care. Whilst there is little evidence of TB transmission outside of migrant communities [49], it remains the case that policies which limit access to healthcare for particular populations have significant implications not only for the health of these individuals but that of the general public. In the case of TB in England, and applicable to other low TB-burden countries where the majority of cases occur among migrants, restricting healthcare access for this population undermines national efforts to eliminate TB. Finally, as the recent UCL-Lancet commission on migration and health highlights, governments have a *“moral and legal obligation”* to uphold the right to the highest attainable standard of health for all people, no matter their immigration status [16].

## Data Availability

Data used in this study is held by Public Health England and was accessed via the London Tuberculosis Register with their permission. The datasets used and/or analysed during the current study are available from the corresponding author on reasonable request with permission from the London Tuberculosis Register.

## Abbreviations

BCG: Bacillus Calmette-Guérin vaccine
CRP: NHS Visitor and Migrant Cost Recovery Programme
LTBI: Latent Tuberculosis Infection
LTBR: London Tuberculosis Register
NHS: National Health Service
TB: Tuberculosis
UK: United Kingdom
